# Emerging Evidence for Three-Dimensional (3D)-Printed Guide Templates in Cervical Spine Trauma Surgery: A Scoping Review

**DOI:** 10.7759/cureus.100164

**Published:** 2025-12-26

**Authors:** Vikash Raj, Aakash Jain, Vishal Kumar, Aman Verma, Pankaj Kandwal, Richa Richa, Sitanshu Barik

**Affiliations:** 1 Orthopaedics, All India Institute of Medical Sciences, Deoghar, Deoghar, IND; 2 Orthopaedics, All India Institute of Medical Sciences, Rishikesh, Rishikesh, IND; 3 Orthopaedics, Postgraduate Institute of Medical Education and Research, Chandigarh, IND; 4 Community and Family Medicine, All India institute of Medical Sciences, Deoghar, Deoghar, IND; 5 Orthopaedics, All India Institute of Medical Sciences, Nagpur, Nagpur, IND

**Keywords:** ‎3d printing, cervical spine fracture, conventional surgery, systematic review, template

## Abstract

Cervical spine fractures are complex injuries that require precise surgical fixation. Conventional freehand pedicle screw placement is associated with prolonged operative time, higher blood loss, and repeated fluoroscopy. Three-dimensional (3D) printing (3DP) offers customized patient-specific templates that may improve safety and efficiency in such cases. This scoping review was conducted in accordance with Preferred Reporting Items for Systematic Reviews and Meta-analyses (PRISMA) 2020 guidelines. PubMed, Embase, Web of Science, and Scopus were searched for comparative studies evaluating 3DP-assisted surgery versus conventional methods in cervical spine fractures. Two reviewers independently screened, extracted data, and assessed study quality using the Methodological Index for Non-Randomized Studies (MINORS) tool. A random-effects model was used for pooled analyses, and heterogeneity was assessed with the I² statistic. Four retrospective comparative studies involving 189 patients were included. Compared with conventional freehand techniques, 3DP-assisted surgery significantly reduced operative time (mean difference: −32.3 minutes; 95%CI −38.6 to −26.0), intraoperative blood loss (mean difference: −121.6 ml; 95%CI −149.0 to −94.1), and fluoroscopy frequency (mean difference: −3.8 shots; 95%CI −4.6 to −2.9). Pain scores at 12 months were also slightly lower in the 3DP group. Heterogeneity ranged from low to moderate, and no major publication bias was identified. 3DP-assisted cervical spine surgery appears to improve operative efficiency and reduce intraoperative burden compared with conventional techniques. These findings represent early, technology-focused evidence supporting the feasibility of 3D-printed guide templates in cervical spine trauma. Given the heterogeneity of injury patterns and anatomical regions, the results should be interpreted as hypothesis-generating rather than definitive.

## Introduction and background

Cervical spine injuries constitute 2-3% of all traumas, and there is an increasing trend towards traumatic fractures of the cervical spine [1-3]. The upper cervical region (occiput, C1, and C2) is more commonly injured than the subaxial spine. However, associated spinal cord injury is more common in the subaxial spine, presumably due to relatively smaller spinal canal diameters. Cervical spine fractures and dislocations often require surgical management in the form of decompression and instrumentation [4-6]. The purpose of surgery is to decompress the spinal cord to improve neurological function and achieve a balanced, stable spine to prevent painful deformity and to facilitate the rehabilitation process by enabling early mobilization.

While two-dimensional imaging is routinely used in spinal surgery, it needs to fully capture the intricacies of the complex three-dimensional (3D) anatomy of the cervical spine and its relationships with adjoining vital structures encountered during the surgery. Consequently, surgeons often resort to prolonged surgery and repeated fluoroscopy to enhance their precision and safety, given the limitations in accuracy and real-time monitoring associated with two-dimensional imaging techniques. In contrast, the adoption of 3D printing (3DP) technology provides surgeons with a more accurate view of the intricate spinal structure and pathological changes. It allows them to virtually experience and plan the surgical procedure on computers [7]. This, in turn, enables the formulation of more suitable preoperative plans and customized templates, guides, or implants, thereby ensuring the safety and effectiveness of the surgery [8,9].

Furthermore, leveraging 3DP technology in spinal surgery offers several additional benefits [10]. By providing patient-specific anatomical models and customized surgical guides, 3DP enhances communication between surgeons and patients or their guardians, enabling clearer explanations of the pathology and planned procedure. These guides also streamline intraoperative execution, thereby reducing the duration of surgery and minimizing the need for repeated fluoroscopic imaging. This results in lower radiation exposure for both medical professionals and patients. Additionally, the improved precision afforded by 3DP helps mitigate intraoperative blood loss and decreases the incidence of complications. Collectively, these advantages contribute to improved surgical outcomes, reinforcing the growing value of 3DP as an effective adjunct in modern spine surgery.

In summary, the emergence and advancement of 3DP technology empower surgical teams to do preoperative planning, explore diverse surgical methods, create custom surgical instruments, and design implants tailored to individual patients' needs, reducing surgical time, intraoperative blood loss, and radiation exposure. The overall outcomes of surgery in terms of improved functional and pain scores and reduced complications have also been reported.

The aim of this review is to synthesize early clinical evidence on the use of 3DP patient-specific drill guide templates in cervical spine trauma surgery. Rather than focusing on a single injury subtype, this review adopts a technology-centred approach, encompassing both upper cervical (atlantoaxial/odontoid) and subaxial injuries, to evaluate the feasibility, safety, and operative efficiency of 3DP-guided instrumentation.

## Review

Methodology

The review was conducted as per the 2020 version of the Preferred Reporting Items for Systematic Reviews and Meta-analyses (PRISMA) guidelines [11]. Given the limited and heterogeneous nature of the available literature, this review is intended to function as a systematic synthesis of early clinical evidence, consistent with principles of a scoping review. The research protocol was registered in the International Prospective Register of Systematic Reviews (PROSPERO) (CRD4202367291).

Search Strategy

Two researchers (SB and AJ) independently conducted a literature search across various electronic databases, namely PubMed, Embase, Web of Science, and Scopus. The search was designed to look for studies focusing on the surgical treatment of cervical spine fractures using 3DP technology. The search keywords used were terms such as "spine trauma", "3D printing", "three-dimensional printing", "additive manufacturing technique", "rapid prototyping", "spine fracture", "cervical spine injury", “cervical spine surgery”, “surgical treatment”, and “surgery”.

The database search results were tabulated in an Excel sheet (Microsoft Corporation, Redmond, Washington, United States), and duplicate studies were identified and removed. Subsequently, the two researchers (SB and AJ) independently screened the search results by going through the titles and abstracts of the identified studies. The studies of interest were identified, while unrelated studies were excluded. The reasons for the exclusion of documents were tabulated. The full texts of the final selected articles were obtained and read independently by both researchers to determine their final eligibility. Any doubt regarding including an article was resolved in consultation with the senior researchers.

Eligibility Criteria

All included studies enrolled adult patients diagnosed with traumatic cervical spine fractures requiring posterior pedicle screw fixation. Patients were included if they underwent either 3DP-assisted pedicle screw placement using patient-specific drill guide templates or conventional freehand screw insertion techniques. Only comparative studies directly evaluating these two approaches were considered. Each study required: (i) Radiologically confirmed cervical spine fracture (C1-C7) suitable for surgical stabilization; (ii) Availability of complete perioperative data (operative time, intraoperative blood loss, and fluoroscopy frequency); and (iii) Minimum follow-up period of 12 months to evaluate postoperative outcomes.

Patients with non-traumatic cervical pathology (tumor, infection, deformity), previous cervical instrumentation, cadaveric or simulated models, and non-comparative case reports or reviews were excluded.

Assessment of Study Quality

The bias in the included studies was evaluated using the Methodological Index for Non-Randomized Studies (MINORS) tool [12]. The MINORS tool evaluates non-randomized studies based on 12 different items. The first eight items are applicable to all studies, while the last four items apply to comparative studies only. Each item is given a numerical value of 0 (no response), 1 (inadequate response), or 2 (adequate response). The final numerical value obtained by adding up the values against each item gives the amount of bias; a higher value indicates less bias.

Data Extraction

Two researchers (SB and AJ) independently extracted relevant demographic and clinical data from the articles finally selected for the review. This data was recorded in Microsoft Excel spreadsheets. In case relevant data were missing from the included articles, an attempt was made to obtain the missing data by mailing the corresponding authors of the respective studies.

The full articles of the studies finally included in the review were read, and data about the name of the study, year of study, place of study, type of study, study duration, study groups, sample size, average age, sex ratio, details of the cervical spine fractures, mean follow up period, the average number of screws inserted, surgical time, amount of blood loss and intra operative fluoroscopy use were extracted and tabulated. Any other data about the outcomes of surgery were also recorded.

Statistical Analysis

Descriptive analysis of all the data extracted from the included studies was performed. Quantitative data were expressed as means, ranges, and standard deviations (SD) and presented in tabular format for clarity. Review Manager (RevMan) (Cochrane, London, United Kingdom) was used for meta-analysis. A random-effects model was applied, and the results were reported as a standardized mean difference (SMD). Heterogeneity was assessed using the I^2^ statistic. Degrees of heterogeneity (low, moderate, and high) were categorized based on I^2^ values of 25%, 50%, and 75%, respectively. A sensitivity analysis was performed to identify any studies contributing to the present heterogeneity. A p-value < 0.05 was taken to be of statistical significance for the overall effect in the Z test. The rest of the data that could not be used in the meta-analysis was presented as a narrative review.

Results

Literature Search

**Figure 1:**
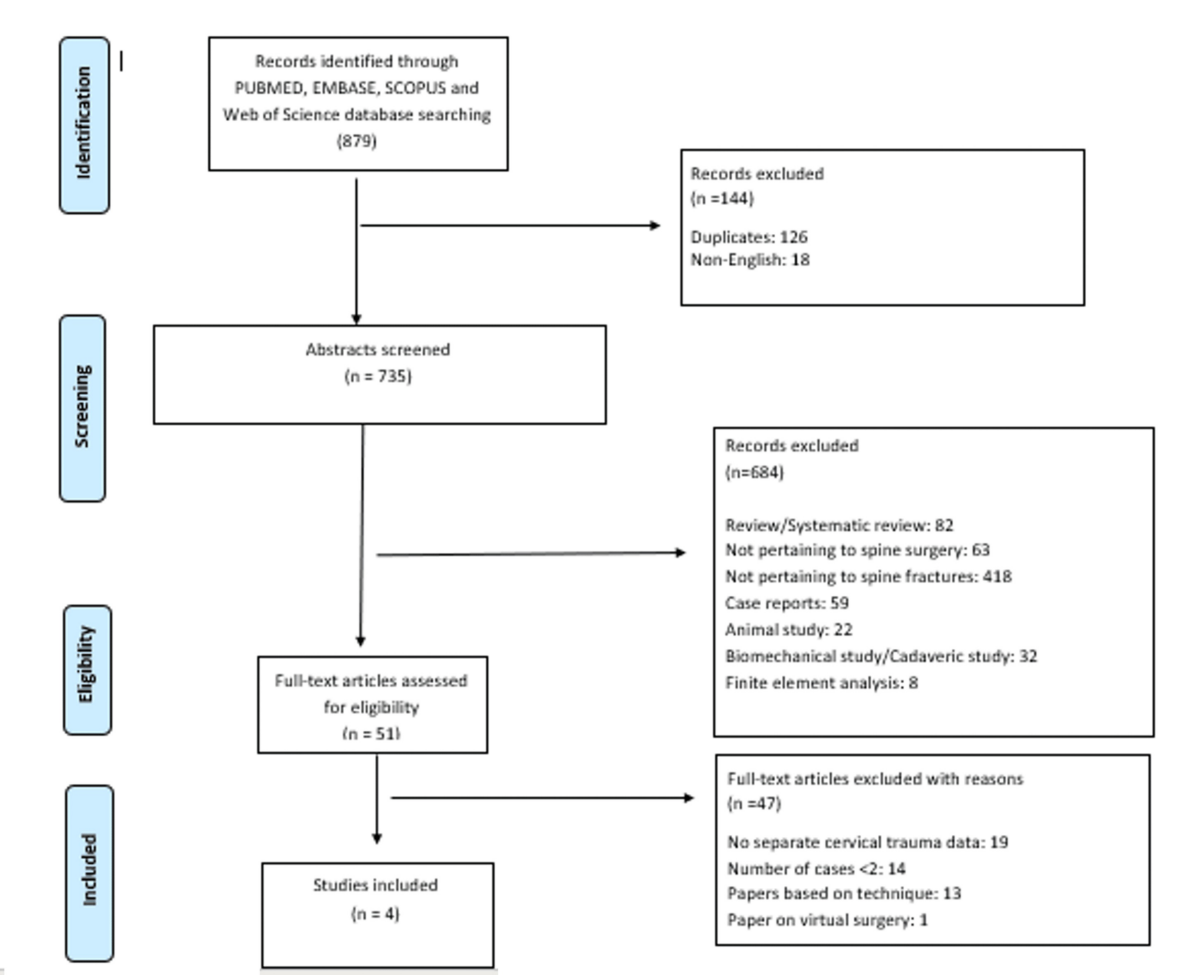
PRISMA flowchart PRISMA: Preferred Reporting Items for Systematic Reviews and Meta-analyses

The database search using the keywords initially showed a total of 879 results. The title and abstract screening was done of 635 studies obtained after excluding duplicates (n = 126) and non-English studies (n 18). Out of the 635 studies, 51 were found eligible for full-text reading. Most of the studies were excluded as they compared the 3DP technique with conventional surgery in patients with fractures not pertaining to the cervical spine (n = 398) and were review articles (n = 54). Other reasons for exclusion were studies not about spine fractures (n = 43), case reports (n = 39), animal studies (n = 12), cadaveric studies (n = 22), and finite element analysis (n = 8). Of the 51 studies subjected to full-text reading, 47 were found ineligible. A total of four studies [13-16] were finally found eligible for inclusion in the study based on the inclusion and exclusion criteria and the PRISMA guidelines (Figure 1).

Study Characteristics

All included studies were non-randomized with relatively small sample sizes, which limits the strength and generalizability of the pooled evidence. All the patients were operated on from a posterior approach, and pedicle screws were used to stabilize the fractured cervical spine. The studies were retrospective without any prospective study sample calculation. The studies demonstrated a low to medium heterogeneity with the I^2^ ranging from 0% to 52%.

All the included studies had a clearly stated aim. All the studies except the one by Li et al. [14] included consecutive patients in the trial, had prospective data collection, and an appropriate follow-up period. The endpoints were aligned with the study's aims, and adequate control groups with baseline equivalence were constituted. No patient was lost to follow-up. The studies had an adequate control group. There was baseline equivalence between the groups, and adequate statistical analysis was performed (Table 1).

**Table 1:** Quality analysis of the studies included based on the MINORS tool MINORS: Methodological Item for Non-Randomized Studies

S No	Study	A clearly stated aim	Inclusion of consecutive patients	Prospective collection of data	Endpoints appropriate to the aim of the study	Unbiased assessment of the study endpoint	Follow-up period appropriate to the aim of the study	Loss to follow up less than 5%	Prospective calculation of the study size	An adequate control group	Contemporary groups	Baseline equivalence of groups	Adequate statistical analyses	Total Score
1	Wu et al., 2023 [13]	2	2	2	2	0	2	2	0	2	2	2	2	20/24
2	Li et al., 2020 [14]	2	0	0	2	0	0	2	0	2	2	2	2	14/24
3	Wang et al., 2019 [15]	1	2	2	2	0	2	2	1	2	0	2	2	18/24
4	Pu et al., 2017 [16]	2	2	2	2	2	2	2	0	2	2	2	2	22/24

Among the four included studies, three [14-16] investigated atlantoaxial and odontoid fractures (C1-C2), whereas one study [13] focused on subaxial cervical spine injuries. Although these represent distinct anatomical regions and injury patterns, all studies evaluated the same surgical adjunct, 3D-printed patient-specific drill guide templates, used to assist pedicle screw placement via a posterior approach.

Demographic Data

There were a total of 189 patients with cervical spine fractures divided into two groups, and the outcomes of surgery using the 3D-printed guides for screw insertion in cervical spine fracture patients (n = 92) were compared with those of conventional techniques for pedicle screw insertion in cervical spine fractures (n = 97) across the four studies (Table 2). All the studies originated in China and were published within the past 10 years. The age of the patients across studies ranged from 22 to 72 years. The male and female ratio was 94:95. All the studies had a follow-up period greater than one year, with the range being 12-45 months. The study by Wu et al. [13] had the smallest sample size of 34 patients, while Li et al.'s study [14] had the largest sample size of 60 patients (Table 2).

**Table 2:** Demographic data of the studies included in the review

Study	Type of study	Diagnosis	Groups		Sample Size		Mean Age		Male-to-Female Ratio		Fracture Characteristics		Follow up	
			Intervention	Control	Intervention	Control	Intervention	Control	Intervention	Control	Intervention	Control		
Wu et al., 2023 [13]	Retrospective study	Cervical spine fracture	3D printed flexible drilling template	3D printed regular drilling template	N = 34		37.9 ± 9.5 years	38.2 ± 6.1 years	12:6	11:5	Cause of Trauma: MVA = 9 High energy fall = 7 Others = 2	Cause of Trauma: MVA = 8 High energy fall = 6 Others = 2	13 months	
					n= 18	n = 16								
Li et al., 2020 [14]	Retrospective study	Odontoid fracture	3D printed navigation template	Free Hand technique	N= 60; As per Grauer Classification: Type II B = 23 Type II C = 37		< 30 years = 13/30 (43%) > 30 years = 17/30 (57%)	< 30 years = 12/30 (40%); >30 years = 18/30 (60%)	9:21	11:19	Fracture Displacement: Anterior = 14/30 (47%) Posterior = 15/30 (50%)	Fracture Displacement: Anterior = 17/30 (57%), Posterior = 9/30 (30%)	NA	
					n = 30	n = 30								
Wang et al., 2019 [15]	Retrospective study	Atlanto axial fracture dislocation	3D printed plate group	Traditional group	N = 46		58 ± 13 years	58 ± 14 years	12:7	14:10	Clinical Diagnosis: (i) Atlantoaxial dislocation = 5, (ii) Atlantoaxial fractures = 5, (iii) Fractures and dislocation of atlantoaxial = 9 Lesions segment : (i) Nonfracture in atlantoaxial = 5, (ii) Atlas fractures = 4, (iii) Axis fracture = 3, (iv) Atlantoaxial fractures = 7	Clinical Diagnosis: (i) Atlantoaxial dislocation = 7, (ii)Atlantoaxial fractures = 8, (iii) Fractures and dislocation of atlantoaxial = 9 Lesions segment : (i) Nonfracture in atlantoaxial = 7; (ii)Atlas fractures = 2; (iii) Axis fracture = 5, (iv) Atlantoaxial fractures = 10	24 ± 11 months	23 ± 9 months
					n = 19	n = 27								
Pu et al., 2017 [16]	Retrospective study	Atlanto axial fracture dislocation	3D printed navigation	Conventional surgery	N = 49		25 – 56 years	22 – 51 years	11:14	14:10	Atlanto axial fracture dislocation = 46 Atlanto axial fracture or disclocation with RA = 3		13.7 months (12 – 45 months)	
					n = 25	n = 24								

Outcomes

**Figure 2:**
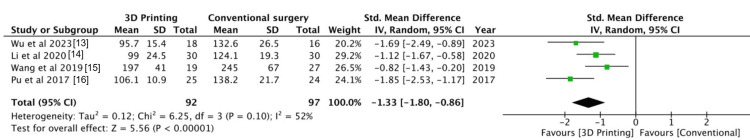
Forest plot comparing the duration of surgery in both groups

**Figure 3:**
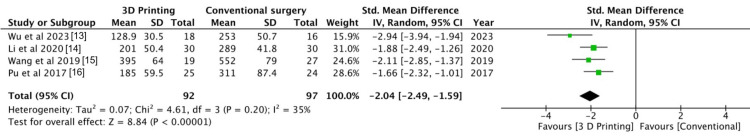
Forest plot comparing intraoperative blood loss in both groups

**Figure 4:**
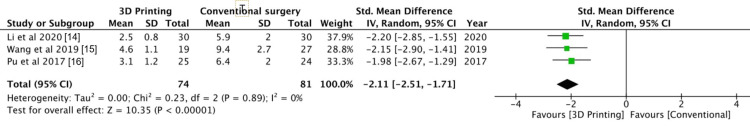
Forest plot comparing fluoroscopy usage in both groups

**Figure 5:**
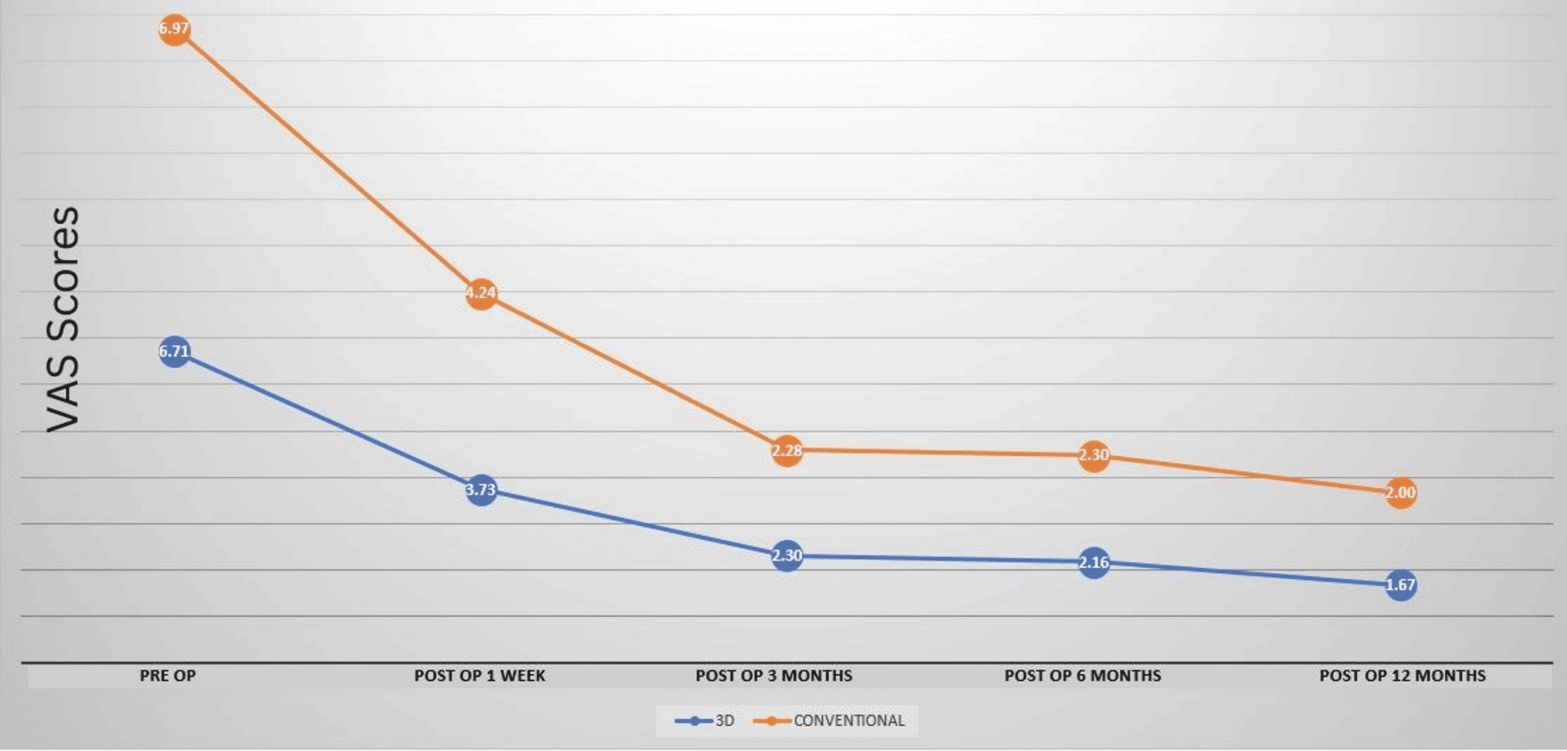
Graphical representation of reduction of VAS scores for pain in both groups VAS: Visual Analog Scale

Owing to clinical heterogeneity across studies with respect to anatomical level, injury type, and control techniques, pooled analyses were performed to explore overall trends rather than to generate injury-specific conclusions. Meta-analysis was performed, and forest plots were constructed to compare the quantitative data, namely (i) duration of surgery, (ii) intraoperative blood loss, and (iii) frequency of intraoperative fluoroscopy use across the four studies. A significant difference in the duration of surgery (SMD -1.33, 95%CI -1.8 to -0.86, Z = 5.56), intra-operative blood loss (SMD -2.04, 95%CI -2.49 to -1.59, Z = 8.84), and fluoroscopy usage (SMD -2.11, 95%CI -2.51 to -1.71, Z = 10.35) was noted across both the groups (Figures 2-4). In absolute terms, a difference of 32.3 minutes (95%CI 26 to 38.6) in surgical time, 121.6 ml (95%CI 94.1 to 149) in intraoperative blood loss, and 3.8 times in fluroscopy shots (95%CI 2.9 to 4.6) was noted among both groups. No publication bias was seen in the funnel plots. The pain scores were lower in the 3DP group (1.67) as compared to the conventional surgery group (2) at 12 months postoperatively (Figure 5). 

Discussion

The primary objective of this study was to explore the role of 3DP technology in managing cervical spine fractures. The research yielded promising results, highlighting the significant benefits that 3DP can bring to cervical spine surgery. Notably, a reduction in surgical time, a decrease in intra-operative blood loss, and a reduced reliance on intra-operative fluoroscopy when utilizing 3DP as a surgical guide were observed [13-16]. Although the included studies involve different cervical regions, the mechanical principles of pedicle screw insertion, the risk of neurovascular injury, and the intended function of 3D-printed drill guides in improving trajectory accuracy are shared across the cervical spine. This provided the rationale for synthesizing these studies within a technology-focused framework. However, these findings are derived from only four non-randomized studies with relatively small sample sizes, which limits the strength and generalizability of the conclusions. These findings are particularly significant because they indicate that the integration of 3DP into cervical spine surgery has the potential to enhance its efficiency and safety, ultimately resulting in improved patient outcomes and positively impacting healthcare provider practices. These early results should therefore be interpreted in comparison with existing adjuncts such as navigation systems and robotics, which also aim to improve accuracy but differ in cost, learning curve, and infrastructure demands.

Advantages of 3DP

One key advantage of 3DP is its ability to provide surgeons with intricate and highly accurate 3D models of a patient's cervical spine. These models are invaluable in aiding in precise preoperative planning and a deeper understanding of the complex anatomical structures within the cervical spine [17]. Moreover, 3DP empowers surgeons to create patient-specific templates, guides, and implants based on these 3DP models. This level of customization enhances surgical precision and can lead to superior surgical outcomes for patients [18]. This study also found that 3DP significantly reduces the need for frequent intraoperative fluoroscopy. This reduction in radiation exposure is beneficial not only for the patients but also for the medical professionals. 3D-printed guides and templates streamline the surgical process, potentially reducing surgical time, which is advantageous for patients and healthcare providers. 3DP models facilitate more precise communication between surgeons and patients or their guardians. This aids in better comprehension of the surgical plans, reducing patient anxiety and improving informed decision-making.

The review of the existing literature underscores the growing interest in 3DP technology in spinal surgery. It also emphasizes the limitations of conventional imaging methods and the demand for more advanced tools to address the complexities of cervical spine surgery. The findings align with previous studies, which have also reported benefits such as reduced surgical time, minimized radiation exposure, and improved patient outcomes, further corroborating the advantages of 3DP in this field [19-23].

While these advantages are notable, similar benefits have been reported with computer-assisted navigation and robotic-assisted systems. Compared to these modalities, 3DP offers the advantage of lower infrastructure requirements and potential cost-effectiveness, though it lacks the real-time adaptability provided by navigation or robotics. Thus, the clinical decision to adopt 3DP should weigh institutional resources, surgeon expertise, and patient needs.

Cost-Benefit and Feasibility Considerations

The adoption of 3DP in cervical spine surgery must be examined not only from a clinical but also from an economic and logistical perspective. Although the present review highlights reduced operative time, blood loss, and fluoroscopy use, the integration of 3DP requires additional resources, including advanced imaging, digital planning software, and access to 3D printers. These factors may limit feasibility in low-resource settings where infrastructure and technical expertise are constrained.

The financial implications of 3DP vary widely. In some reports, the cost of producing a patient-specific guide or model ranges between USD 150-500, depending on material and local availability [20,23]. While this represents an added upfront expense, potential downstream savings may arise from shortened operative duration, reduced complication rates, and decreased reliance on intraoperative fluoroscopy, which collectively reduce operating room costs and radiation exposure [19]. Studies in lumbar and deformity surgery suggest that these efficiencies can offset the initial cost of printing, particularly in high-volume centers [24]. However, such evidence remains sparse in cervical trauma, and cost-effectiveness analyses specific to this indication are urgently required.

Feasibility also extends to the learning curve associated with 3DP planning and execution. Unlike navigation and robotic systems, which require specialized consoles and capital investments, 3DP may offer a more accessible alternative for institutions unable to acquire expensive platforms [22]. Nevertheless, variability in accuracy and reproducibility between centers remains a challenge, underscoring the need for standardized protocols and surgeon training.

A critical feasibility concern in cervical spine trauma is the time required to design and manufacture patient-specific 3D-printed drill guide templates. Most cervical spine fractures requiring surgical stabilization are ideally managed within 24-48 hours, particularly in the presence of neurological compromise. In the studies included in this review, the reported workflow typically involved preoperative CT acquisition, digital planning, and in-house 3DP, allowing guide fabrication within 24-72 hours, depending on institutional infrastructure and printing capacity. Consequently, the application of 3D-printed guides may be most suitable for hemodynamically stable patients, delayed fixation cases, or injuries without urgent neurological deterioration, rather than for all emergency cervical trauma presentations. It is also noteworthy that all four included studies were conducted in tertiary-care university hospitals, where access to advanced imaging, digital planning software, and on-site or affiliated 3DP facilities was readily available. Such infrastructure may not be universally accessible, particularly in non-academic or resource-limited settings, which may limit the generalizability of these findings.

In summary, while 3DP demonstrates promise in enhancing surgical safety and efficiency, its adoption must be weighed against practical barriers of cost, resource allocation, and institutional capacity. Economic analyses and multicenter feasibility studies will be essential in establishing its role in routine cervical spine practice.

Implications for the Future

The future of cervical spine surgery is highly promising. The adoption of 3DP technology could revolutionize surgical practices in this domain. It opens doors to further innovations, including developing advanced surgical instruments, tailored implants, and techniques customized to individual patient needs. Additionally, reducing surgical time and complications could lead to improved patient outcomes and cost savings in healthcare. Future research should broaden the scope of 3DP applications in spinal surgery, exploring its potential in other spinal regions and conducting long-term evaluations of its benefits [13,25,26].

Despite short-term benefits such as reduced operative time and radiation, evidence on long-term outcomes such as fusion rates, implant survival, complication profiles, and patient-reported functional outcomes remains absent. Studies in other spinal regions, such as lumbar deformity correction and tumor resection, have shown durable benefits of 3DP, but whether these translate to cervical pathology requires further evaluation.

3DP as a Cost-Effective Alternative to Navigation and Robotics

While computer navigation and robotic-assisted systems have improved the precision of screw placement and reduced complications in cervical spine surgery, their widespread use remains constrained by high acquisition costs, steep learning requirements, and dependence on real-time imaging infrastructure [21,27]. In many low- and middle-income settings, such barriers make these technologies impractical. In contrast, 3D-printed drill guide templates provide a surgeon-friendly, reproducible, and economical solution that allows accurate instrumentation without continuous intraoperative imaging. Our review demonstrates that the use of 3DP can significantly reduce operative time, intraoperative blood loss, and fluoroscopy exposure, offering both clinical and occupational benefits. Moreover, as the technology matures, its integration into standard practice could represent a scalable and sustainable bridge between conventional freehand methods and advanced robotic platforms. This positions 3DP as a valuable adjunct in resource-limited settings and a driver of safer, more efficient cervical spine surgery.

Strengths and Limitations

This study boasts notable strengths, including a systematic approach aligned with PRISMA guidelines, adherence to the MINORS tool for quality assessment, and the inclusion of a meta-analysis to provide quantitative support for the advantages of 3DP in cervical spine surgery. Nevertheless, the evidence base remains preliminary. The review included only four small, non-randomized studies, all originating from China, which substantially limits the robustness, external validity, and generalizability of the conclusions. Furthermore, the studies' retrospective nature and non-randomized design could impact the overall quality of the evidence. The inclusion of sole English language studies could have potentially missed out appropriate studies from this review. 

A key limitation of this review is the heterogeneity of included studies, including differences in anatomical region (atlantoaxial versus subaxial spine), injury patterns, and control techniques. Consequently, the findings cannot be generalized to all cervical spine injuries. The results should be interpreted as early, hypothesis-generating evidence supporting the feasibility of 3D-printed guide templates rather than as definitive comparative effectiveness data.

Another important limitation relates to the turnaround time required for 3DP guide production, which may delay surgery in acute trauma scenarios. Additionally, all included studies originated from academic university centres with ready access to 3DP infrastructure, limiting the applicability of these findings to non-tertiary or resource-constrained environments. These factors must be carefully considered before extrapolating the routine use of 3D-printed guides in emergency cervical spine trauma.

## Conclusions

This study underscores the tremendous potential of 3DP technology to enhance the outcomes of cervical spine surgery. The findings suggest that 3DP can reduce surgical time, minimize blood loss, and decrease radiation exposure, offering substantial benefits to both our patients and us as healthcare providers. This review highlights the emerging role of 3D-printed guide templates in cervical spine trauma surgery and serves as a foundation for future, injury-specific comparative studies. Robust multicenter randomized trials are required before definitive clinical recommendations can be made.
